# Protein Profiling Identifies Biomarkers for Predicting Disease Severity in Anti-NMDAR Encephalitis

**DOI:** 10.3390/ijms27177577

**Published:** 2026-08-24

**Authors:** Shufang Zhao, Fang Xu, Lili Cui, Weibi Chen, Gang Liu, Huimin Zhang, Dawei Shan, Shuting Chai, Le Yang, Guoliang Chai, Dongshan Wan, Yan Zhang

**Affiliations:** 1 Department of Neurology, Xuanwu Hospital Capital Medical University, National Center for Neurological Disorders, Beijing 100053, China; zhaoshu_fang@126.com (S.Z.); xvfangtcm@163.com (F.X.); samantha47@163.com (L.C.); chenweibi@126.com (W.C.); liugangqingyi@sina.com (G.L.);; 2 Institute of Sleep and Consciousness Disorders, Beijing Institute of Brain Disorders, Collaborative Innovation Center for Brain Disorders, Capital Medical University, Beijing 100069, China

**Keywords:** autoimmune encephalitis, anti-NMDAR encephalitis, proximity extension assay (PEA), protein biomarkers

## Abstract

Anti-N-methyl-D-aspartate receptor (NMDAR) encephalitis is a severe autoimmune neurological disorder characterized by pathogenic antibodies against the NMDAR. A systematic protein profiling approach is warranted to identify biomarkers capable of predicting disease status. An Olink proximity extension assay (PEA) profiled 91 inflammation-related proteins from anti-NMDAR encephalitis patients. Disease severity or prognosis were assessed by CASE score or mRS score at 6-month follow-up. Patients were stratified into distinct molecular clusters using unsupervised clustering. Logistic regression models incorporating selected biomarkers were developed to predict disease severity and prognosis, followed by absolute quantification using ELISA. Patients were classified into four consensus clusters. Clusters 1 and 2 corresponded to the mild group, while Cluster 3 represented the severe group, consistent with CASE score above 6. Cluster 4 showed heterogeneous clinical features. Elevated serum levels of IL-10, IL-6, and SIRT2, as well as increased CSF levels of CXCL10, CXCL11, and MMP10, were positively associated with severe disease. Conversely, several proteins including LTA and CCL11, CCL8, TGFB1, CXCL6 were associated with severe disease or unfavorable 6-month outcomes. A logistic regression model combining serum CXCL6 and CCL11 with CSF MMP10 achieved an area under the curve (AUC) of 0.95 for predicting disease severity. Serum CCL11 alone showed predictive value for 6-month prognosis, with an AUC of 0.79. These findings delineate distinct protein signatures associated with clinical heterogeneity of anti-NMDAR encephalitis. Prediction models incorporating multiple biomarkers may provide an approach for disease severity stratification and prognosis forecast.

## 1. Introduction

Autoimmune encephalitis (AE) comprises a broad spectrum of inflammatory disorders of the central nervous system (CNS), characterized by pathogenic autoantibodies targeting neuronal cell-surface or intracellular proteins [1,2]. Anti-N-methyl-D-aspartate receptor (NMDAR) encephalitis is associated with the most common autoantibody identified among all discovered antibodies associated with AE, characterized by IgG autoantibodies targeting the GluN1 subunit [3]. Patients typically present with an acute or subacute clinical course involving a broad range of neurological and psychiatric manifestations, including seizures, psychiatric symptoms, movement disorders, impaired consciousness, and autonomic dysfunction [4]. The annual incidence of antibody-confirmed AE is approximately 2.1 per million, as reported in France in 2018 [5]. However, anti-NMDAR encephalitis can result in severe neurological dysfunction and life-threatening complications, with 50–75% of patients requiring intensive care unit (ICU) admission [6,7]. First-line immunotherapies including intravenous immunoglobulin (IVIG), corticosteroid pulse therapy, and plasma exchange (PE) are effective for most patients. Second-line agents such as rituximab and cyclophosphamide can be considered for treatment-resistant cases [8]. Importantly, even among patients with severe AE, timely diagnosis and appropriately tailored immunotherapy can improve clinical outcomes [6,8].

For the diagnosis of anti-NMDAR encephalitis, the presence of IgG antibodies against the GluN1 subunit of the NMDAR with at least one characteristic clinical symptom is sufficient to establish a diagnosis [4]. In addition, several clinical and laboratory parameters, including MRI, EEG, and inflammatory cytokines/chemokines, have been reported to predict disease severity and outcome for anti-NMDAR encephalitis [9]. For example, the One-Year Functional Status (NEOS) score was established to predict 1-year prognosis based on factors including ICU admission, time to treatment, 4-week treatment response, MRI abnormalities, and cerebrospinal fluid (CSF) white blood cell count [10]. Although the NEOS score provides valuable prognostic information, its calculation requires clinical response data collected 4 weeks after treatment initiation, limiting its application for early assessment.

In addition, cytokine/chemokine profiles of anti-NMDAR encephalitis have been well investigated, with a particular focus on CSF IL-6, IL-10, IL-17, CXCL10, and CXCL13 levels [9,11,12,13]. Some of these proteins may contribute to disease pathogenesis [14]. For example, elevated IL-17 can promote differentiation of T helper (Th)17 cells, while CCL20/22 recruit them into the CNS, thereby potentially exacerbating disease [15]. However, these studies have relied on the single-plex assay, providing a limited perspective on individual mediators. Furthermore, these biomarkers lack sufficient disease specificity, as similar alterations may also occur in other infectious or inflammatory CNS disorders [16]. However, distinct disease states may generate distinguishable signatures characterized by coordinated changes across multiple proteins [17]. Therefore, comprehensive profiling approaches are warranted to systematically characterize inflammation-related protein patterns and evaluate their associations with clinical phenotypes in anti-NMDAR encephalitis. To address this, several studies using bead-based multiplex immunoassays have demonstrated distinct protein signatures in patients with anti-NMDAR encephalitis compared with other inflammatory CNS diseases or healthy controls [18,19]. However, most of these studies did not establish clinically applicable models for predicting disease status. Moreover, analytical performance, particularly sensitivity and limits of detection, can vary across assay platforms and manufacturers, potentially limiting the clinical translation of these findings [20].

The proximity extension assay (PEA) has emerged as a highly sensitive and scalable platform for multiplex protein profiling [2,21]. By combining dual-antibody recognition with DNA-based signal amplification and PCR or next-generation sequencing readout, PEA enables proteome-level profiling using minimal sample volumes and has been widely applied to protein biomarker discovery and the development of clinically relevant protein signatures [22].

Here, we used the Olink PEA to profile a 91-plex panel of inflammatory protein biomarkers in both serum and CSF from patients with anti-NMDAR encephalitis. We aimed to characterize protein signatures, investigate their associations with clinical severity and outcomes, and develop multivariable prediction models incorporating candidate biomarkers, which could inform timely clinical management decisions for anti-NMDAR encephalitis, and provide potential candidates for further investigation as therapeutic targets that can be quantified using routine laboratory assays.

## 2. Results

### 2.1. Patient Categorization by Unsupervised Clustering and Clinical Score

We performed a 91-plex PEA to profile serum and CSF samples from 36 patients with anti-NMDAR encephalitis. After excluding proteins for which ≥25% of samples were below the limit of detection (LOD) and those lacking evidence of extracellular distribution, 104 proteins in serum or CSF were retained for analysis (serum 58/91, CSF 46/91; Supplementary Table S1).

**Figure 1:**
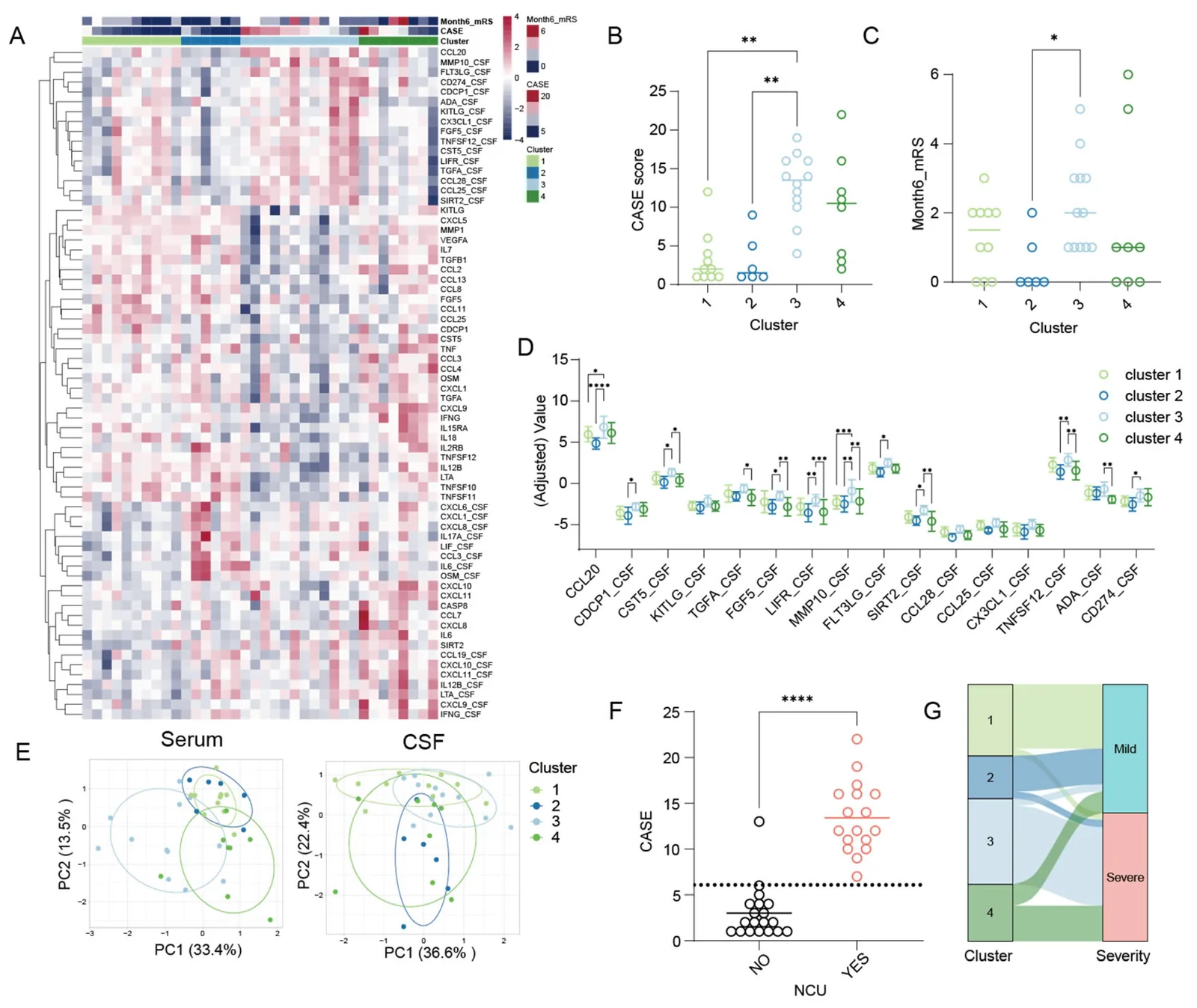
Unsupervised consensus clustering of anti-NMDAR encephalitis patients corresponded to CASE score-based severity classification. (**A**) The consensus clustering analysis separated the samples into four clusters. The heatmap hierarchically clustered the distinct protein profile of each cluster (adjusted *p* < 0.1). (**B**,**C**) Differences between 4 clusters in CASE score (**B**) and 6-month mRS score (**C**). Median scores are shown. (**D**) Comparison of the upregulated protein signature of Cluster 3 relative to the other three clusters. Error bars are presented as the mean ± SD. (**E**) PCA of protein profiles in serum or CSF for 4 clusters. (**F**) Difference in CASE score between patients admitted and not admitted into NCU. The horizontal dashed line indicates the maximum score of 6 for patients who were categorized into the mild group. Median scores are shown. (**G**) Alluvial diagram reveals the correspondence between consensus clustering and severity groupings. *p*-values in (**B**,**C**) were calculated using Kruskal–Wallis test with Dunn’s multiple comparisons test. *p*-value in (**F**) was calculated using Mann–Whitney U test. *p*-values in (**D**) were calculated using two-way ANNOVA with Turkey’s multiple comparisons test. **** *p* < 0.0001, *** *p* < 0.001, ** *p* < 0.01, and * *p* < 0.05.

Unsupervised consensus clustering was performed based on PEA results. Consensus matrices showed distinct clustering patterns when the number of clusters (k) was set to 3 or 4 (Supplementary Figure S2A,B). The empirical cumulative distribution function (CDF) curves and corresponding changes in the area under the CDF curves were further evaluated. The relative change in CDF area showed an elbow at k = 4, beyond which further increases in k resulted in only marginal changes, supporting the selection of 4 clusters (Figure 1A; Supplementary Figure S2C–E).

Cluster 3 was associated with higher Clinical Assessment Scale for Autoimmune Encephalitis (CASE) scores than Clusters 1 and 2 and higher 6-month modified Rankin Scale (mRS) scores than Cluster 2 (Figure 1B,C). In contrast, Cluster 4 showed substantial heterogeneity, with both high and low CASE and 6-month mRS scores. Compared with the other clusters, Cluster 3 exhibited a widespread reduction in serum protein levels, whereas serum CCL20 was increased (Figure 1A,D; Supplementary Figure S2F). In CSF, MMP10 levels were markedly higher in Cluster 3 than in the other 3 clusters, whereas most other CSF proteins showed only increasing trends (Figure 1D). Cluster 4 was characterized by increased levels of several proteins, including serum IL-6, CXCL10, and CXCL11, as well as CSF CXCL10, CXCL11, LTA, and IFN-γ. In Cluster 2, which generally had lower CASE scores, CSF levels of IL-6 and IL-17A were increased (Figure 1A; Supplementary Figure S2G). Principal component analysis (PCA) demonstrated distinct serum protein profiles between Cluster 3 and Clusters 1 or 2, whereas the separation between clusters was less apparent in CSF (Figure 1E). Collectively, these findings provide preliminary evidence that protein profiles capture molecular heterogeneity associated with disease severity in anti-NMDAR encephalitis.

Among the 36 anti-NMDAR encephalitis patients enrolled in this study, all neurological intensive care unit (NCU) patients had CASE scores above 6. In contrast, only 1 non-NCU patient (5.6%) had score above 6 (Figure 1F). The results from the consensus clustering method corresponded well with severe and mild patient groups classified using 6 as a threshold. Clusters 1 and 2 predominantly comprised patients with CASE scores of 6 or less, whereas all patients in Cluster 3 had CASE scores greater than 6. In contrast, Cluster 4 showed heterogeneous CASE scores and could not be clearly assigned to either group (Figure 1G). These findings supported the use of a CASE score greater than 6 to define the severe disease group in subsequent analyses. Regarding 6-month functional outcomes, clusters 1 and 2 predominantly comprised patients with good outcomes (6-month mRS score ≤ 2), whereas no clear correspondence between cluster assignment and poor outcome (6-month mRS score > 2) was observed for Clusters 3 and 4 (Supplementary Figure S2H).

### 2.2. Patient Clinical Characteristics

Among all patients, 18 patients (50%) were classified as severe, while 18 (50%) were classified as mild. Patients with severe symptoms were younger on average (*p* = 0.021), had longer hospital stays (*p* < 0.001), higher NCU admission rates (*p* < 0.001), and higher rates of respiratory failure requiring ventilator support (*p* = 0.001). For the core symptoms of anti-NMDAR encephalitis, except for cognitive decline (*p* = 1), severe patients were more likely to show consciousness decline (*p* < 0.001), speech impairment (*p* = 0.003), seizures (*p* = 0.044), psychiatric symptoms (*p* = 0.007), movement disorders (*p* < 0.001), autonomic disorders (*p* < 0.001). In examinations, severe patients had relatively higher titers of serum anti-NMDAR antibody titers (*p* = 0.021) and were more likely to show abnormalities on MRI (*p* = 0.044). In subsequent treatments, severe patients received more intensive interventions: corticosteroid pulse treatment (*p* = 0.045), IVIG (*p* = 0.018), PE (*p* = 0.001), and immunosuppressive agents (*p* = 0.086) (Table 1).

A total of 28 patients (77.8%) had a good outcome, while 8 (22.2%) had poor outcomes. Patients with poor outcomes had higher CASE scores at onset (*p* = 0.023). Poor-outcome patients showed no significant differences in age (*p* = 0.183) or gender (*p* = 1). However, they had longer hospital stays (*p* = 0.054) and higher NCU admission rates (*p* = 0.016). Except for consciousness decline (*p* = 0.016), other core symptoms did not show significant associations with outcome. Regarding treatment, poor-outcome patients were more likely to receive PE (*p* = 0.013) (Supplementary Table S2).

### 2.3. Correlation Analysis of Inflammation-Related Proteins

Among the proteins profiled, CCL20, SIRT2, IL-10, and HGF in serum were significantly positively correlated with CASE scores. Serum levels of several proteins, including CCL11, TGFB1, CCL8, and TNF superfamily members (LTA, TNFSF10, and TNFSF11), showed the strongest negative correlations with CASE scores (Supplementary Figure S3A,C).

Fewer proteins were significantly correlated with the 6-month mRS score. Serum SIRT2, IL-17C, and CCL20 and CSF FLT3LG were positively correlated with the 6-month mRS score, whereas serum TNFSF10 and KITLG were negatively correlated (Supplementary Figure S3A,D).

These correlations were generally of medium intensity, with absolute Spearman correlation coefficients ranging from 0.3 to 0.7. Compared with protein levels showing significant positive correlations with CASE or 6-month mRS scores, those showing significant negative correlations showed stronger intercorrelations. The protein–protein correlation analysis revealed a highly interconnected network among the 18 proteins inversely correlated with CASE or 6-month mRS scores. In contrast, the seven additional proteins negatively associated with clinical scores displayed relatively weak correlations both within this subgroup and with the other negatively correlated proteins (Supplementary Figure S3B,E).

### 2.4. Differences in Inflammation-Related Protein Levels According to Disease Severity and Clinical Outcome

**Figure 2:**
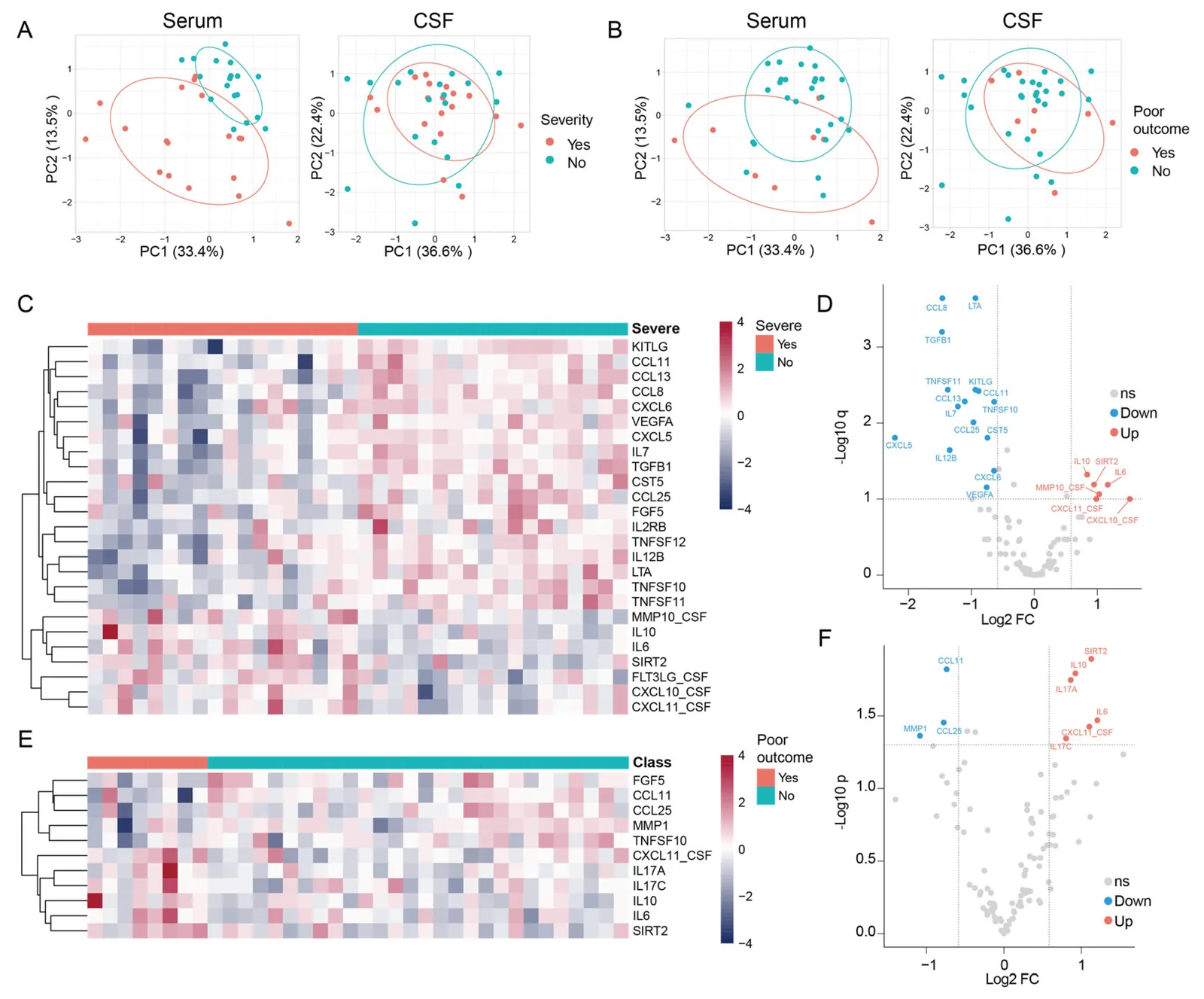
Differences in inflammation-related protein levels by disease severity and outcome in anti-NMDAR encephalitis. (**A**,**B**) PCA of proteins in serum or CSF in patients grouped by clinical severity (**A**) or 6-month outcome (**B**). (**C**,**D**) Heatmap or volcano plot (adjusted *p* < 0.1) of significantly different protein levels between mild and severe groups. (**E**,**F**) Heatmap or volcano plot of significantly different protein levels between good and poor-outcome groups (nominal *p* < 0.05). *p*-values were calculated by *t*-test followed by Benjamini–Hochberg multiple-comparison correction.

PCA revealed distinct serum protein profiles between severe and mild anti-NMDAR encephalitis, as classified by the CASE scores, with the mild group showing less interindividual variability. In contrast, no clear separation between the severe and mild groups was observed in the CSF protein profiles (Figure 2A). When patients were stratified according to the 6-month mRS scores, no clear separation between the good- and poor-outcome groups was observed in either serum or CSF protein profiles (Figure 2B).

A differential protein analysis according to disease severity showed significantly increased levels of serum IL-6, IL-10, and SIRT2 and CSF CXCL10, CXCL11, and MMP10 in patients with severe disease (adjusted *p* < 0.1, fold change > 1.5). A larger number of proteins showed decreased levels in the severe group (adjusted *p* < 0.1; fold change > 1.5), particularly serum CCL8, TGFB1, and LTA (Figure 2C,D).

In patients with poor outcomes, serum IL-17A, IL-17C, IL-6, IL-10, and SIRT2 and CSF CXCL11 were significantly increased, while serum FGF5, CCL11, CCL25, MMP1, and TNFSF10 were downregulated (Figure 2E,F).

For exploratory biomarker selection, proteins showing between-group differences at a nominal *p* < 0.05 were retained as candidate biomarkers for subsequent univariable and multivariable analyses of disease severity or 6-month outcome.

### 2.5. Regression Model for Predicting Severity in Anti-NMDAR Encephalitis

We next sought to facilitate the translation of PEA-derived biomarkers to conventional single-plex assays, such as ELISA, and to develop a model for predicting clinical severity in patients with anti-NMDAR encephalitis. However, normalized protein expression (NPX) values generated by PEA represent relative rather than absolute protein concentrations, and analytical sensitivity and detection ranges may differ across assay platforms. To address this issue, each continuous biomarker was dichotomized as “high” or “low” using the optimal cutoff determined by maximizing the Youden index on the receiver operating characteristic curve (ROC) curve (Supplementary Table S3). The resulting binary variables were subsequently used for prediction model development. Candidate biomarkers were then quantified by ELISA to determine the corresponding cutoff values in absolute concentration units. This approach enabled the translation of PEA-derived prediction models to conventional assays for clinical use, provided that the corresponding absolute concentration cutoffs fell within the analytical range of the selected assays.

**Figure 3:**
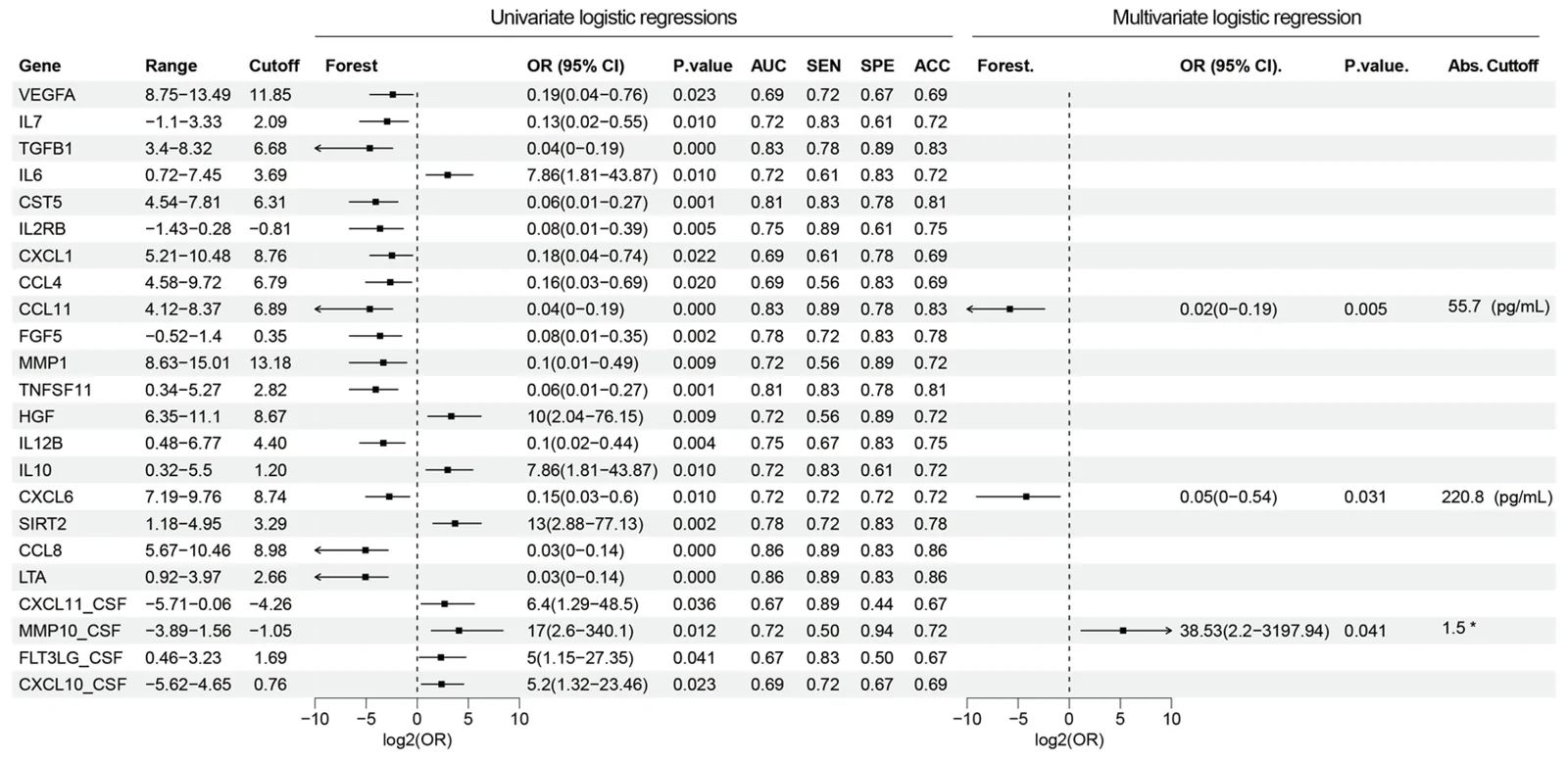
Forest plot of univariable and multivariable logistic regression analyses of dichotomized biomarkers associated with disease severity. * MMP10_CSF (pg/mL)/CSF total protein (mg/dL). Abbreviations: OR: odds ratio; AUC: area under the curve; SEN: sensitivity; SPE: specificity; ACC: accuracy.

After being converted to binary variables, candidate biomarkers were subjected to univariate logistic regression analyses with clinical severity, yielding 23 candidates with significant associations which were further included in multivariate logistic regression analysis (Figure 3). After systematically evaluating all possible biomarker combinations, we found that the 2-biomarker model combining CCL11 and LTA achieved performance comparable to the 3-biomarker model (Supplementary Table S4). However, subsequent ELISA measurements showed that LTA concentrations were quantifiable in only a small proportion of patients (7/36). Linear regression between NPX values and the available ELISA measurements yielded an estimated absolute cutoff concentration of 0.62 pg/mL, which was below the lower limit of the ELISA assay (2.2 pg/mL), limiting the clinical applicability of the CCL11/LTA-based model (Supplementary Figure S4). Based on the predefined selection criteria, we finally selected the combination of serum CCL11 (OR, 0.02; 95% CI, 0–0.19), serum CXCL6 (OR, 0.05; 95% CI, 0–0.54), and CSF MMP10 (OR, 38.53; 95% CI, 2.20–3197.94), which demonstrated comparable predictive performance while incorporating a CSF biomarker that may provide a more direct reflection of CNS inflammation. This combination was subsequently used to establish the multivariable logistic regression model (Figure 3).

**Figure 4:**
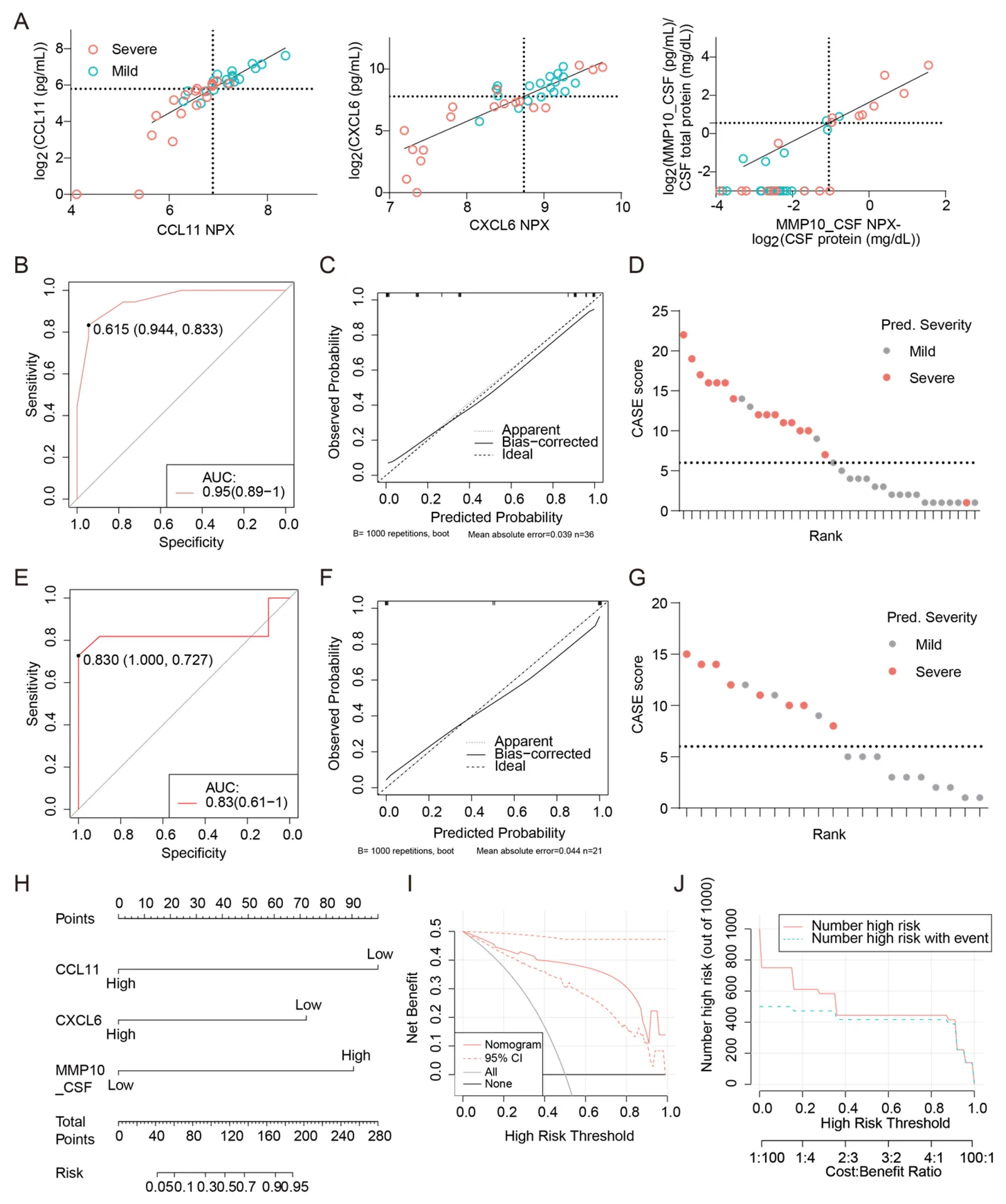
Selection of biomarkers for predicting disease severity in anti-NMDAR encephalitis. (**A**) Fitting curves show that the absolute concentrations quantified by ELISA have a linear relationship with NPX values obtained by PEA for CCL11, CXCL6, and MMP10_CSF in the training set. The dashed lines represent the cutoffs. Values undetectable by ELISA are set to 0 for CCL11 and CXCL6, and -3 for MMP10_CSF on the Y-axis. (**B**) ROC curve of the logistic regression model for predicting clinical severity with 3 biomarkers (CCL11, CXCL6, and MMP10_CSF) in the training set. The point indicates the optimal threshold. The 95% CI was calculated using the DeLong methods. (**C**) The calibrate curve showed the goodness of fit using the HL method in the training set. (**D**) The waterfall diagram shows the predicted severe and mild status in the validation set. The dashed line represents the CASE score threshold of 6. (**E**) ROC curve of logistic regression model to predict clinical severity with 3 biomarkers (CCL11, CXCL6 and MMP10_CSF) in the validation set. The point indicates the optimal threshold. 95% CI was calculated by DeLong methods. (**F**) The calibrate curve showed the goodness of fit by HL method in the validation set. (**G**) Waterfall diagram of the prediction for severe and mild status. The dashed line represents the CASE score threshold of 6 in the training set. (**H**) The nomogram of the model shows the predicted scores of the three dichotomous biomarkers: CXCL6, CCL11, and MMP10_CSF, and the corresponding predicted risks of severe status based on the total score. (**I**,**J**) DCA and CIC of the three-biomarkers model.

To determine the absolute values of these three biomarkers, ELISA detection was performed. The ELISA analyses showed lower LOD compared to PEA. NPX values linearly fitted with the log2 scale of ELISA values, allowing the mapping of absolute cutoffs for the three biomarkers (CCL11: 55.7 pg/mL, CXCL6: 220.8 pg/mL, MMP10_CSF: 1.5, MMP10_CSF/CSF total protein) (Figure 4A). The ROC curve indicated that the model area under the curve (AUC) was 0.95 (95% CI: 0.89–1), sensitivity was 0.83, and specificity was 0.94 (Figure 4B; Supplementary Table S4). Internal validation indicated that the average AUC of the model was 0.90 (95% CI: 0.75–1), and accuracy was 0.84 (95% CI: 0.67–1). Hosmer–Lemeshow (HL) testing confirmed the model’s goodness of fit (*p* = 0.99). Observed and predicted probability curves showed consistency with the ideal situation (Figure 4C). In addition, high accuracy (0.89) was observed (Figure 4D).

To evaluate the robustness of the model, the external validation was used for performance assessment (Supplementary Table S5). The ROC curve showed that the model AUC was 0.83 (95% CI: 0.61–1), sensitivity was 0.73, specificity was 1, and accuracy was 0.86 (Figure 4E–G).

Next, a nomogram was constructed of the prediction model to determine severe probability separately (Figure 4H). The predicted risk threshold set to 0.6 provided the greatest benefit in predicting patients’ severity (Figure 4I,J).

### 2.6. Regression Model Established for Predicting 6-Month Outcomes in Patients with Anti-NMDAR Encephalitis 

Similar to disease severity, considering that patients with good outcomes after treatment accounted for the majority, serum CCL11 (OR: 0.02, 95% CI: 0–0.19) alone was enough to predict outcomes with a relatively high accuracy of 0.89, a specificity of 0.964, and a sensitivity of 0.625 (Supplementary Figure S5; Supplementary Table S3). Absolute levels of CCL11 in serum assayed by ELISA had a linear fit with corresponding NPX values, allowing mapping of the absolute cutoff for CCL11 (24.6 pg/mL) (Supplementary Figure S6A). The ROC curve showed that the model AUC was 0.79 (95% CI: 0.61–0.98) (Supplementary Figure S6B). Internal validation showed that the average AUC was 0.81 (95% CI: 0.59–1), and accuracy was 0.88 (95% CI: 0.67–1). HL testing confirmed the model’s goodness of fit (*p* > 0.999). Observed and predicted probability curves showed good consistency with the ideal situation (Supplementary Figure S6B). In addition, high accuracy was observed (Supplementary Figure S6C). For validating the model, the ROC curve showed the model AUC was 0.76 (95% CI: 0.57–0.96), sensitivity was 0.78, specificity was 0.75, and accuracy was 0.76 (Supplementary Figure S6D–F).

## 3. Discussion

In this study, we employed PEA to simultaneously profile 91 inflammation-related proteins in the serum and CSF of 36 patients with anti-NMDAR encephalitis. Unsupervised clustering identified four distinct protein-profile clusters, among which Cluster 3 closely corresponded to severe disease as defined by a CASE score greater than 6. Several inflammatory proteins were elevated in patients with severe disease, including CSF CXCL10, CXCL11, and MMP10 and serum IL-6, IL-10 and SIRT2. Conversely, reduced levels of several proteins, including the TNF superfamily member LTA, CCL11, CCL8, TGFB1, and CXCL6, were associated with greater disease severity or unfavorable 6-month prognosis. Based on these findings, serum CXCL6 and CCL11 together with CSF MMP10 were incorporated into a multivariable logistic regression model for predicting disease severity. In addition, serum CCL11 alone showed predictive value for 6-month functional outcome.

Interestingly, IL-17 was not associated with clinical severity but rather clinical outcome. IL-17 is a major effector cytokine of Th17 cells and contributes to inflammatory responses and disruption of the blood–brain barrier (BBB) integrity, potentially facilitating the entry of circulating immune mediators into the CNS [23]. Previous studies have suggested that persistent Th17-related immune responses may contribute to sustained neuroinflammation and unfavorable outcomes in AE [15,24]. In our study, elevated serum IL-17 together with CCL20, a chemokine involved in the recruitment of CCR6-expressing Th17 cells, may therefore reflect persistent peripheral Th17-associated immune activation and potentially contribute to long-term neurological outcomes.

Some non-conventional inflammatory proteins were identified, particularly MMP10 in CSF, and SIRT2 in serum. MMP10 belongs to the matrix metalloproteinases (MMP) family and plays critical roles in extracellular matrix degradation/remodeling as well as immunity [25]. The elevated MMP10 in CSF was found in brain white matter disease and correlated with radiographic and clinical severity [26]. Increased CSF levels of MMP10 in mild cognitive impairment subjects showed a higher probability of Alzheimer’s disease and a faster cognitive decline [27]. Together with our finding that CSF MMP10 was elevated in patients with severe anti-NMDAR encephalitis, these observations suggest that MMP10 may serve as a marker of CNS tissue remodeling or injury.

SIRT2 is predominantly an intracellular NAD+-dependent deacetylase with emerging immunoregulatory functions. Mechanistically, SIRT2-mediated deacetylation of p70S6K suppresses IL-2 production while promoting IL-17 release from CD4+ T cells and Th17 differentiation [28]. Elevated circulating SIRT2 has been reported in patients with acute ischemic stroke or classical Parkinson’s disease [29,30]. Although the biological significance of circulating SIRT2 remains incompletely understood, its elevation in patients with severe anti-NMDAR encephalitis may reflect altered immune regulation and warrants further mechanistic investigation.

In our study, serum showed greater between-group differences than CSF, suggesting that CNS inflammation reflected by CSF may not fully account for disease severity or prognosis. Severe anti-NMDAR encephalitis can lead to various complications, such as pneumonia, sepsis, systemic inflammatory response syndrome (SIRS), organ failure, or other complications [31,32]. These complications may exacerbate the clinical course and contribute to unfavorable outcomes, especially in vulnerable patients [33]. Therefore, systemic inflammatory responses secondary to severe neurological disease and its associated complications may partly account for the dysregulation of inflammatory proteins observed in serum, including elevated IL-6 and IL-10 levels.

The divergent changes observed between CSF and serum may also reflect, at least in part, compartment-specific immune response and redistribution of circulating immune cells. Alterations in the neutrophil-to-lymphocyte ratio (NLR) have been observed across various CNS diseases and may serve as an indicator of disease severity [33,34]. Recruitment of peripheral lymphocytes across the BBB is an important component of CNS autoimmunity, and chemokines such as CXCL10 and CXCL11 can promote trafficking of activated T cells into inflamed tissues. Conversely, severe CNS injury can induce systemic immunological alterations through activation of the hypothalamic-pituitary-adrenal axis and sympathetic nervous system. Such neuroimmune responses have been well characterized after stroke, where increased glucocorticoids and catecholamines are associated with lymphopenia, altered leukocyte function, and peripheral immunosuppression [35]. These mechanisms provide a potential framework for interpreting the reduced serum levels of several chemokines and immune mediators observed in our study, including CCL8, CCL11, CXCL5, CXCL6, and members of the TNF superfamily [36,37].

Several limitations of this study should be acknowledged. First, protein profiling was restricted to a predefined 91-plex inflammation-related panel in serum and CSF from 36 patients. More comprehensive protein profiling using larger PEA panels may provide a broader representation of disease proteomes. The predictive performance of inflammatory biomarkers for long-term functional outcome was more modest than that for disease severity. Larger longitudinal cohorts are therefore needed to clarify their prognostic value and to determine whether these biomarkers are associated with treatment responses, particularly rituximab. The relatively small sample size poses an important challenge for prediction model development and increases the risk of model overfitting. We therefore adopted a parsimonious conventional logistic regression approach rather than more complex machine-learning algorithms to improve the generalizability of the models. However, larger datasets may also enable the application of machine-learning approaches and integration of additional data modalities, such as neuroimaging and electrophysiological features, to characterize more complex relationships between molecular profiles and clinical phenotypes.

Second, inflammatory protein alterations may not be specific to anti-NMDAR types of AE. Although overlapping cytokine and chemokine profiles have been reported across different subtypes of AE and other inflammatory CNS disorders, disease-specific differences may also exist [38,39,40]. Future studies incorporating other AE types and inflammatory CNS diseases will therefore be necessary to determine the specificity and broader applicability of the protein signatures and prediction models identified in this study.

Collectively, our findings reveal distinct inflammation-related protein signatures associated with the clinical heterogeneity of anti-NMDAR encephalitis and identify a candidate biomarker panel for early severity stratification and outcome prediction. They provide a molecular perspective on the complex interplay between peripheral and CNS immune responses and may facilitate a more individualized assessment of disease severity and prognosis.

## 4. Materials and Methods

### 4.1. Participants

**Figure 5:**
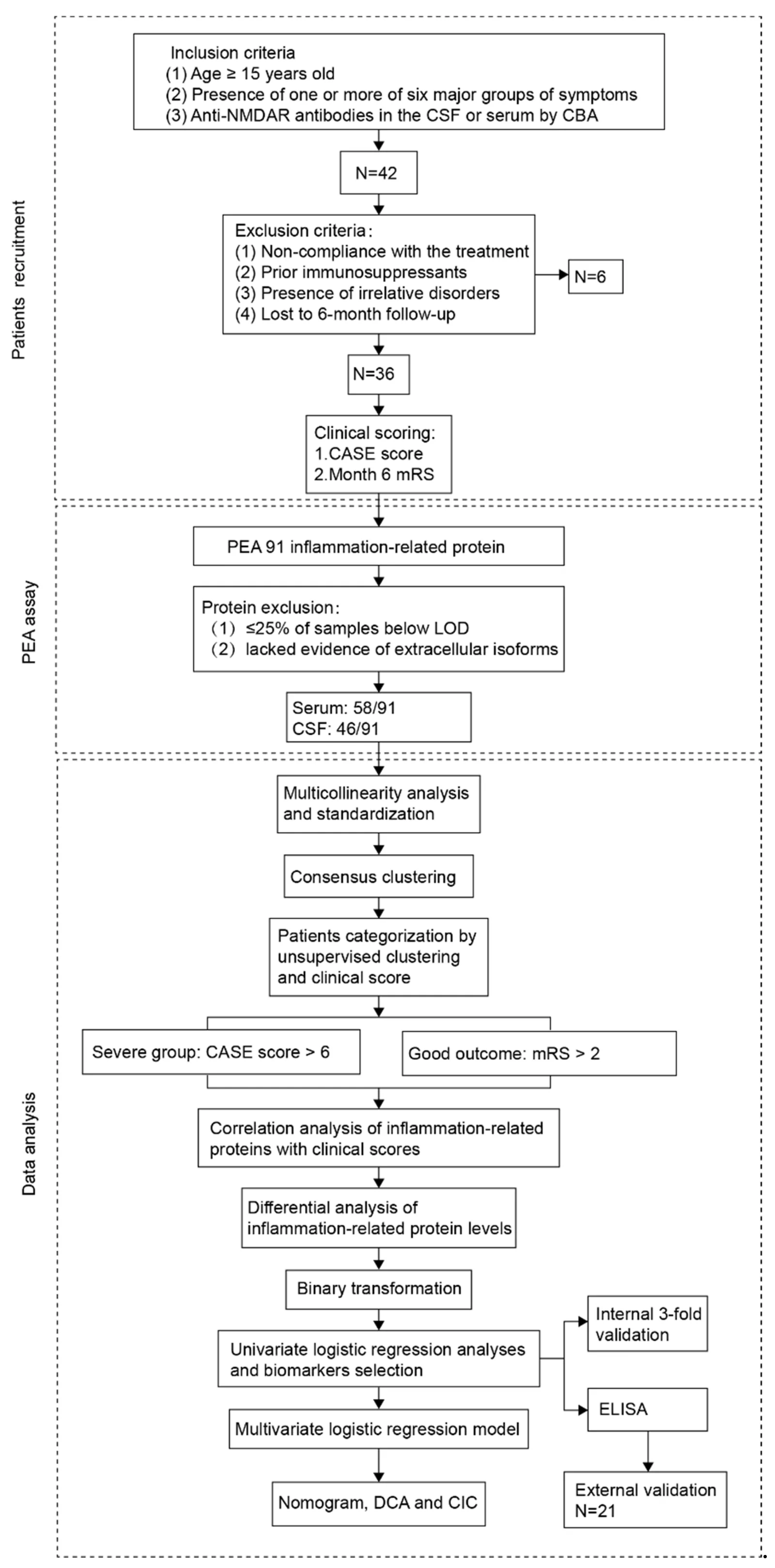
Work flowchart. Abbreviations: CASE: Clinical Assessment Scale for Autoimmune Encephalitis; CBA: cell-based assay; LOD: limit of detection; DCA: decision curve analysis; CIC: clinical impact curves.

The study workflow is illustrated in Figure 5. A total of 36 patients with anti-NMDAR encephalitis who were treated at the Department of Neurology, Xuanwu Hospital, Capital Medical University were recruited for PEA-based protein profiling. Samples from an additional 21 patients were prospectively collected for temporal external validation of the model. All patients fulfilled the inclusion criteria for anti-NMDAR encephalitis detailed below [4]: (1) age ≥ 14 years; (2) presence of one or more of six major groups of symptoms of abnormal behavior or cognitive dysfunction, speech dysfunction, seizures, movement disorders, decreased level of consciousness, autonomic dysfunction, or central hypoventilation; (3) anti-NMDAR antibodies in the CSF or serum by cell-based assay. Exclusion criteria were as follows: (1) poor adherence to treatment; (2) prior treatment with immunosuppressive agents; (3) presence of unrelated disorders including brain tumors, metabolic diseases, drug poisoning, and systemic autoimmune disease; (4) loss to follow-up before the 6-month assessment. Patients who were admitted to the NCU met at least one of the following criteria: respiratory failure requiring mechanical ventilation, impaired consciousness (GCS ≤ 12), status epilepticus, or mRS > 3. Patients’ serum or CSF samples were collected within 7 days after admission or transfer to the NCU before immunotherapy.

### 4.2. Clinical Scoring

CASE scoring was used to assess clinical severity in the active phase of AE. CASE was developed in 2019 as a more precise scale for evaluating severity in AE compared to the mRS. CASE includes nine items: seizures, memory dysfunction, psychiatric symptoms, consciousness, language problems, dyskinesia/dystonia, gait instability and ataxia, brainstem dysfunction and muscle weakness. Each item is scored from 0 to 3, with a maximum total score of 27 [41].

The patients were followed up at 6 months after enrollment through on-site or telephone interviews with patients and their relatives. The mRS was used to evaluate the patients’ functional outcome and an mRS score above 2 at 6 months after enrollment was defined as a poor outcome. The mRS was originally developed to scale global disability after stroke, with scores ranging from 0 to 5 based primarily on the ability to walk and functional independence, with a score of 6 representing patient death [42].

### 4.3. PEA

Protein concentrations in the serum and CSF from patients with anti-NMDAR encephalitis were quantified using PEA with the commercial Target 96 Inflammation Panels (developed by Olink Proteomics, Shanghai, China; Supplementary Table S1), following the manufacturer’s protocol [43]. Briefly, immunoassays were established by coupling designed oligonucleotide sequences to protein-targeting antibodies. Only when two antibodies simultaneously recognized a protein, their attached oligonucleotides hybridized complementarily in proximity and were pre-extended by DNA polymerase. The PCR sequences were then subjected to quantitative real-time PCR (qPCR) detection using a Dynamic Array IFC on the BioMark system (Fluidigm, South San Francisco, CA, USA). The data generated from qPCR were normalized using inter-plate controls (IPC) and converted to NPX on a log2 scale. This NPX served as the required input file for subsequent bioinformatics analysis workflows.

For quality control, each reaction on the chip included four types of internal quality controls (2 immune controls, 1 extension control, and 1 detection internal control) to monitor the entire experimental process. External quality control involved eight samples (2 positive sample controls, 3 negative controls, and 3 IPCs). Confirmation of PEA assay stability required an intra-plate coefficient of variation (CV) of ≤15% and an inter-plate CV of ≤25% based on the control samples.

Only proteins for which fewer than 25% of samples had measurements below the LOD were included in the analysis. For measurements below the LOD, the corresponding raw extended values were retained for analysis. Intracellular or transmembrane proteins without established evidence of extracellular soluble or exosomal forms were excluded because their detection in biofluids may result from cellular debris. Protein measurements in CSF showed greater multicollinearity than those in serum (Supplementary Figure S1A–D). Thus, total CSF protein concentration was used to adjust CSF protein measurements. Specifically, the log2-transformed total CSF protein concentration (mg/dL) was subtracted from each protein’s NPX value to obtain an adjusted CSF protein level (Supplementary Figure S1E).

### 4.4. ELISA

Absolute quantification of proteins was performed using ELISA kits (Multisciences, Hangzhou, China or Elabscience, Wuhan, China) following the manufacturer’s protocol. Optical density (OD) values at 450 nm were read using a Synergy^TM^ Neo2 instrument (Biotek, Winooski, VT, USA).

### 4.5. Data Analysis and Statistics

All statistical analyses and data visualization were performed using R software (version 4.2.2) with custom R scripts, IBM SPSS Statistics (version 26.0; IBM Corp., Armonk, NY, USA), and GraphPad Prism (version 10; GraphPad Software, Boston, MA, USA).

Data preprocessing and manipulation were performed using the tidyverse package (version 2.0.0). Consensus clustering was performed to group samples based on their protein expression profiles by utilizing ConsensusClusterPlus package (version 1.76.0) [44], with the following parameters: bootstrap: 1000, sampling proportion: 0.8, clustering method: “Partitioning around medoids”, distance: Pearson correlation.

PCA was performed using the stats::prcomp() function. To discover differential protein levels between anti-NMDAR encephalitis groups, significances were calculated using one-way ANOVA or Student’s *t*-test followed by Benjamini–Hochberg multiple-comparison tests. Statistical significance of differences in protein levels among clusters was assessed using two-way ANOVA followed by Tukey’s multiple-comparisons test. Statistical significance was assessed using the R package rstatix (version 0.7.2). Heatmaps were generated using the pheatmap() function. PCA plots and volcano plots were generated using the ggplot() function.

To analyze baseline patient characteristics, the Wilcoxon rank-sum test was used for ordinal variables. Fisher’s exact tests were used for categorical variables. Multi-correlation analyses among inflammatory proteins or with clinical scores utilized corrplot::corrplot() function. Correlation network analysis was conducted using Cytoscape software (version 3.8.2). Demographic and clinical characteristics were summarized using the tableone::CreateTableOne() function.

Significant differential proteins between groups with different disease severities or outcomes (nominal *p* < 0.05) were identified as candidate biomarkers for model construction. The pROC::roc() function was used to calculate the AUC. The cutoff value for each biomarker was determined at the maximum Youden index (Sensitivity + Specificity − 1) on the ROC. The included biomarkers were then transformed into binary forms (high and low) based on the previously calculated cutoffs and then subjected to univariate logistic model calculation. Only those with *p* < 0.05 were candidates in multivariate logistic regression modeling. Univariable and multivariable logistic regression models were constructed using the stats::glm() function. Forest plots were generated using the forestploter::forest() function.

Possible combinations of candidate biomarkers were screened to construct multivariate logistic regression models. The Akaike information criterion (AIC), AUC, *p*-values (<0.05) of each variable, and accuracy were calculated for each model. The HL test and pseudo-R-squared were used to evaluate the goodness of fit of the model using SPSS. Several factors were considered when selecting the optimal biomarker combinations for multivariate logistic regression modeling: (1) models with superior predictive performance were preferred; (2) biomarkers with absolute concentrations that could be readily measured using clinically feasible assays, such as ELISA, were favored; and (3) CSF-derived proteins positively correlated with disease severity were prioritized because they may more directly reflect pathological processes within the CNS. Internal validation of models was conducted by 200 iterations of a 3-fold cross-validation method.

For the selected model predicting clinical severity or 6-month outcome, a clinical nomogram and HL calibration plots were generated using the rms:: nomogram() function. Decision curve analysis (DCA) and clinical impact curves (CIC) were performed using the rmda::decision_curve().

## Figures and Tables

**Table 1:** Association of clinical characteristics with severity of anti-NMDAR encephalitis.

		Mild (*n* = 18)	Severe (*n* = 18)	*p*			Mild (*n* = 18)	Severe (*n* = 18)	*p*
CASE score		2.00 [1.00, 3.75]	12.50 [11.00, 16.00]	<0.001	CSF protein, mg/dL		36.00 [23.00, 45.75]	24.00 [18.00, 42.00]	0.125
6-month mRS		1.00 [0.00, 2.00]	1.50 [1.00, 3.00]	0.023	CSF anti-NMDAR titer (%)	1:10	7 (43.8)	2 (16.7)	0.106
Gender (%)	F	6 (33.3)	10 (55.6)	0.315		1:32	9 (56.2)	10 (83.3)	
	M	12 (66.7)	8 (44.4)			1:100	2 (11.1)	6 (33.3)	
Age, years		36.50 [30.00, 52.75]	26.50 [19.00, 38.75]	0.021	Serum anti-NMDAR titer (%)	0	10 (55.6)	3 (16.7)	0.021
Inpatient, days		10.50 [8.25, 12.75]	28.50 [24.00, 49.50]	<0.001		1:10	4 (22.2)	12 (66.7)	
NCU (%)	Yes	0 (0.0)	17 (94.4)	<0.001		1:32	4 (22.2)	3 (16.7)	
	No	18 (100.0)	1 (5.6)			1:100	0 (0.0)	0 (0.0)	
NCU, days		0.00 [0.00, 0.00]	28.00 [15.75, 49.50]	<0.001	MRI abnormal (%)	Yes	13 (72.2)	6 (33.3)	0.044
Movement disorder (%)	Yes	0 (0.0)	9 (50.0)	0.001		No	5 (27.8)	12 (66.7)	
	No	18 (100.0)	9 (50.0)		Combined Ab (%)	GABAR	1 (5.6)	0 (0.0)	0.735
Consciousness decline (%)	Yes	0 (0.0)	17 (94.4)	<0.001		GFAP	1 (5.6)	0 (0.0)	
	No	18 (100.0)	1 (5.6)			MOG	1 (5.6)	1 (5.6)	
Speech impairment (%)	Yes	1 (5.6)	10 (55.6)	0.003		No	15 (83.3)	17 (94.4)	
	No	17 (94.4)	8 (44.4)		Combined tumor (%)	No	17 (94.4)	15 (83.3)	0.603
Seizure (%)	Yes	6 (33.3)	13 (72.2)	0.044		Teratoma	1 (5.6)	3 (16.7)	
	No	12 (66.7)	5 (27.8)		IVMP treatment (%)	Yes	13 (72.2)	18 (100.0)	0.045
Psychiatric symptom (%)	Yes	9 (50.0)	17 (94.4)	0.007		No	5 (27.8)	0 (0.0)	
	No	9 (50.0)	1 (5.6)		IVIG treatment (%)	Yes	6 (33.3)	14 (77.8)	0.018
Movement disorder (%)	Yes	1 (5.6)	12 (66.7)	<0.001		No	12 (66.7)	4 (22.2)	
	No	17 (94.4)	6 (33.3)		PE treatment (%)	Yes	0 (0.0)	9 (50.0)	0.001
Autonomic nervous involved (%)	Yes	0 (0.0)	10 (55.6)	<0.001		No	18 (100.0)	9 (50.0)	
	No	18 (100.0)	8 (44.4)		MMF (%)	Yes	4 (22.2)	10 (55.6)	0.086
Cognition decline (%)	Yes	9 (50.0)	10 (55.6)	1		No	14 (77.8)	8 (44.4)	
	No	9 (50.0)	8 (44.4)		Rituximab (%)	Yes	9 (32.1)	3 (37.5)	1
CSF pressure, mmH_2_O		162.50 [120.00, 197.50]	195.00 [150.00, 252.50]	0.064		No	19 (67.9)	5 (62.5)	
					Relapse in 6 months (%)	Yes	0 (0.0)	1 (5.6)	1
CSF cells, 10^6^ cells/L		6.50 [2.00, 36.00]	9.00 [5.25, 17.00]	0.495		No	18 (100.0)	17 (94.4)	

Consecutive or ordinal variables are shown in median [IQR]. Abbreviations: IQR = interquartile range; NCU = neurological intensive care unit; CSF = cerebrospinal fluid; IVIG = intravenous immunoglobulin; PE = plasma exchange; IVMP = intravenous methylprednisolone; NMDAR = N-methyl-D-aspartate receptor; GABAR = gamma aminobutyric acid receptor; GFAP = glial fibrillary acidic protein; MOG = myelin oligodendrocyte glycoprotein; MMF = Mycophenolate Mofetil.

## Data Availability

The original contributions presented in this study are included in the article/Supplementary Materials. Further inquiries can be directed to the corresponding authors.

## Supplementary Materials

The following supporting information can be downloaded at: https://www.mdpi.com/article/10.3390/ijms27177577/s1.
