# Supplementary material for: Protein Profiling Identifies Biomarkers for Predicting Disease Severity in Anti-NMDAR Encephalitis
Source: Int J Mol Sci. 2026 Aug 24;27(17):7577. doi: 10.3390/ijms27177577 (PMC13566394; doi:10.3390/ijms27177577)
Supplement: Supplementary file 1 [file ijms-27-07577-s001.zip › ijms-4421139-supplementary.pdf]

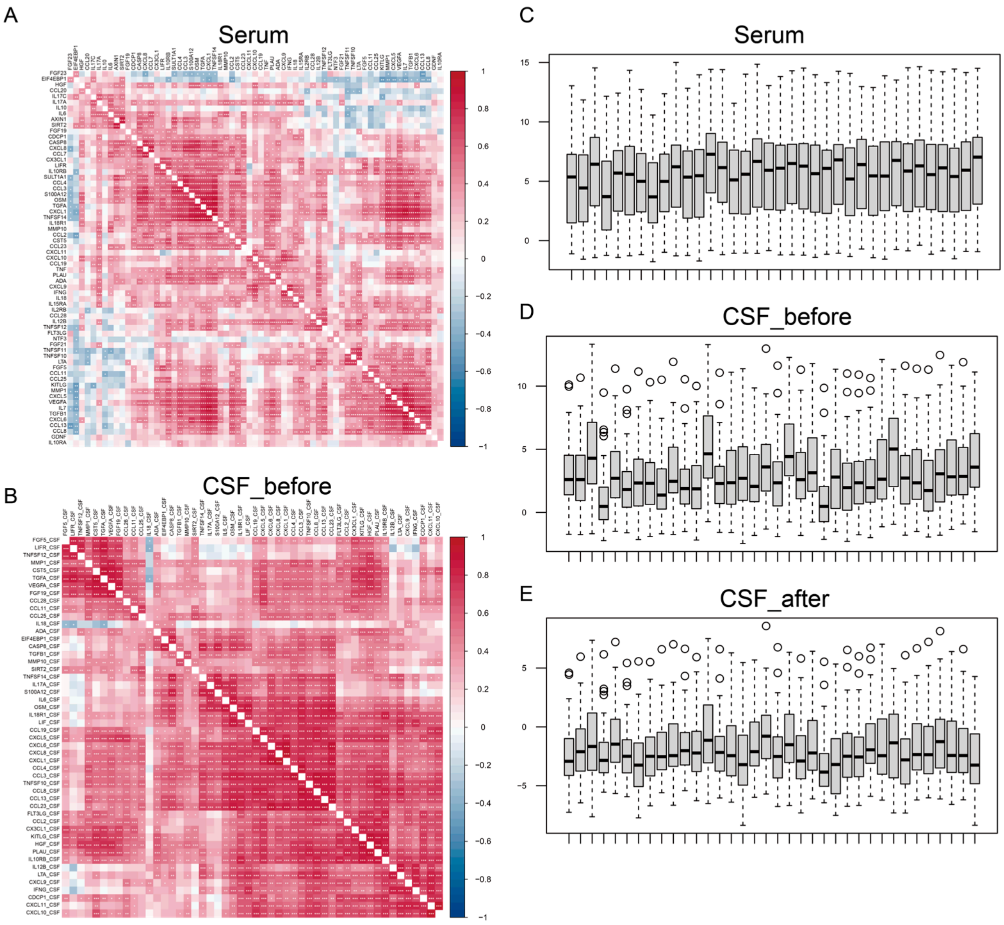

Supplementary Figure S1. Multicollinearity in serum or CSF across anti-NMDAR encephalitis.

(A and B) Correlation matrix of protein levels in serum (A) and CSF (B). The scale bar represents Pearson's r.

(C and D) Boxplot shows the distribution of serum (C) and CSF (D) protein levels across patients.

(E) Boxplot shows the distribution of CSF protein levels across patients after adjusted by CSF total protein.

****p < 0.0001, ***p < 0.001, **p < 0.01 and *p < 0.05.

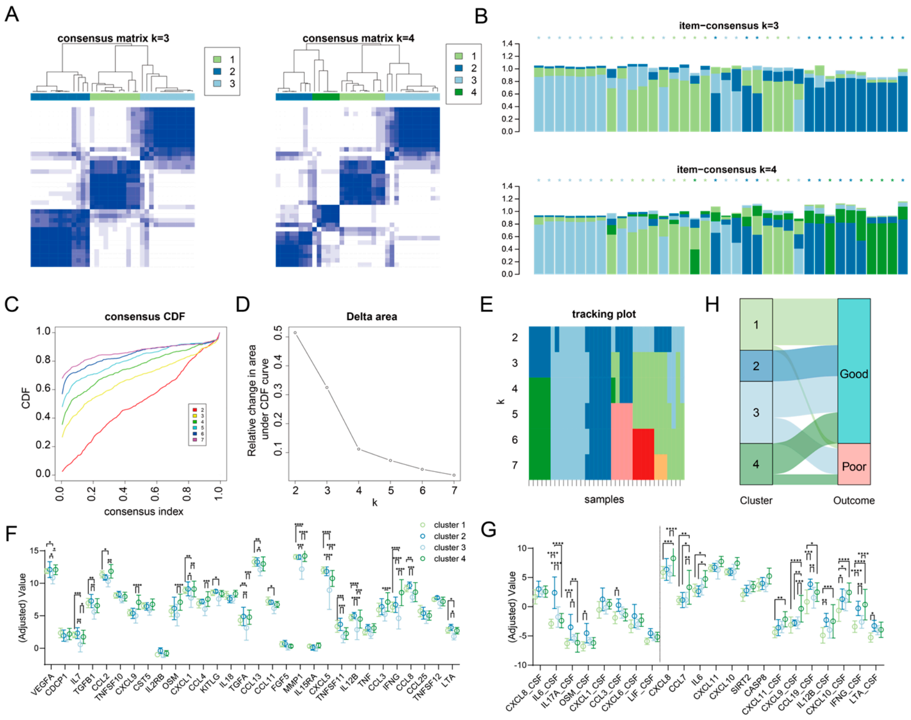

Supplementary Figure S2. Consensus clustering of anti-NMDAR encephalitis.

(A) The consensus matrix.

(B) The item-consensus analysis shows the proportional representation of each cluster within individual samples.

(C) Consensus CDF.

(D) Relative change in the area under CDF.

(E) The tracking plot shows changes in sample clustering when k is incrementally increased from 2 to 7.

(F and G) Comparison of downregulated protein signature of Cluster 3 (F) and upregulated protein signatures of Cluster 1, 2 (G) relative to the other three clusters. Error bars are mean ± SD.

(H) Alluvial diagram reveals the correspondence between consensus clustering and outcome groupings.

p-values in (F and G) were calculated by two-way ANNOVA with Turkey's multiple comparison test. ****$p < 0.0001$, ***$p < 0.001$, **$p < 0.01$ and *$p < 0.05$.

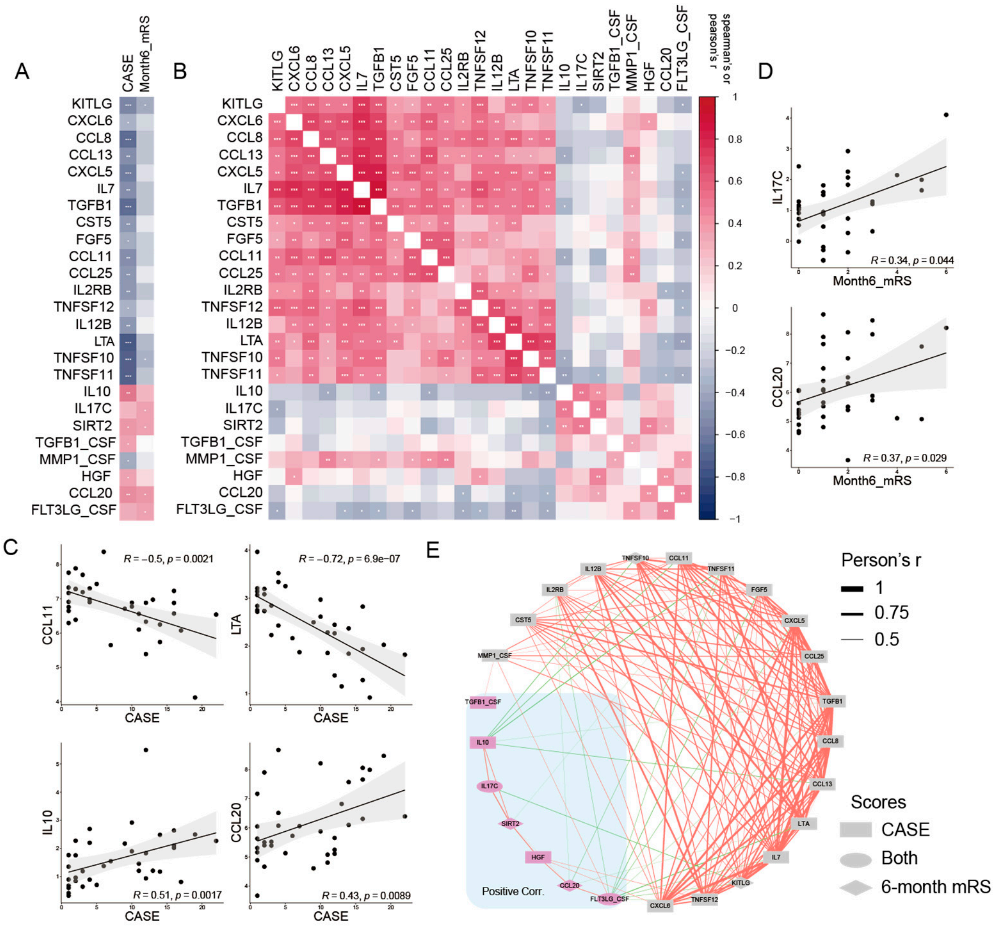

Supplementary Figure S3. Correlation of inflammation-related protein levels with clinical severity and prognosis scores in anti-NMDAR encephalitis.

(A) Levels of inflammation-related proteins in serum and CSF that were significantly correlated with CASE score or 6-month mRS score ($p < 0.05$).

(B) Correlation matrix of clinically significant relevant protein levels. The scale bar represents Spearman's r (A) and Pearson's r (B). ****$p < 0.0001$, ***$p < 0.001$, **$p < 0.01$ and *$p < 0.05$.

(C) Scatter plots shows the Spearman's correlation and p-value test between CASE score and CCL20, LTA, IL-10, CCL11 in serum.

(D) Scatter plots showing the Spearman's correlation and p-value test between 6-month mRS score and CCL20, IL-17C in serum.

(E) Correlation network of clinically relevant proteins. Red lines represent positive correlations and green lines represent negative correlations. Line thickness represents Pearson's r. Only Pearson's $r > 0.5$ and p-value < 0.05 are shown.

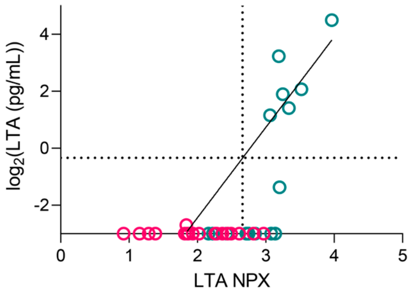

Supplementary Figure S4. Curve fitting demonstrated the relationship between absolute LTA concentrations quantified by ELISA and PEA-derived NPX values. Dash lines represent the cutoffs. Values undetectable by ELISA are set to -3.

			Univariate logistic regression							
Gene	Range	Cutoff	Forest	OR (95% CI)	P.value	AUC	SEN	SPE	ACC	Abs. Cuttoff
IL17C	−0.63−4.1	1.18		14.78(2.18−298.89)	0.018	0.78	0.88	0.68	0.78	
IL17A	−1.78−3.49	−0.57		11(1.98−90)	0.011	0.77	0.75	0.79	0.78	
CCL11	4.12−8.37	6.17		0.02(0−0.19)	0.002	0.79	0.62	0.96	0.89	24.6 (pg/mL)
FGF5	−0.52−1.4	0.23		0.09(0.01−0.5)	0.011	0.77	0.75	0.79	0.78	
MMP1	8.63−15.01	12.50		0.08(0.01−0.52)	0.012	0.71	0.50	0.93	0.83	
IL10	0.32−5.5	2.38		13(1.93−121.05)	0.012	0.71	0.50	0.93	0.83	
SIRT2	1.18−4.95	3.68		13.8(2.42−116.43)	0.006	0.79	0.75	0.82	0.81	
CCL25	2.63−6.95	4.82		0.09(0.01−0.5)	0.011	0.77	0.75	0.79	0.78	
CXCL11_CSF	−5.71−0.06	−2.94		7.67(1.43−49.26)	0.021	0.72	0.62	0.82	0.78	

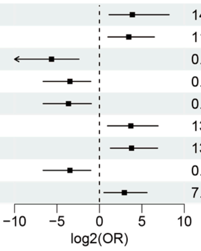

Supplementary Figure S5. Forest plot of univariable logistic regression analyses of dichotomized biomarkers associated with clinical outcomes.

Abbreviations: OR: Odds ratio; AUC: Area under curve; SEN: Sensitivity; SPE: Specificity; ACC: Accuracy.

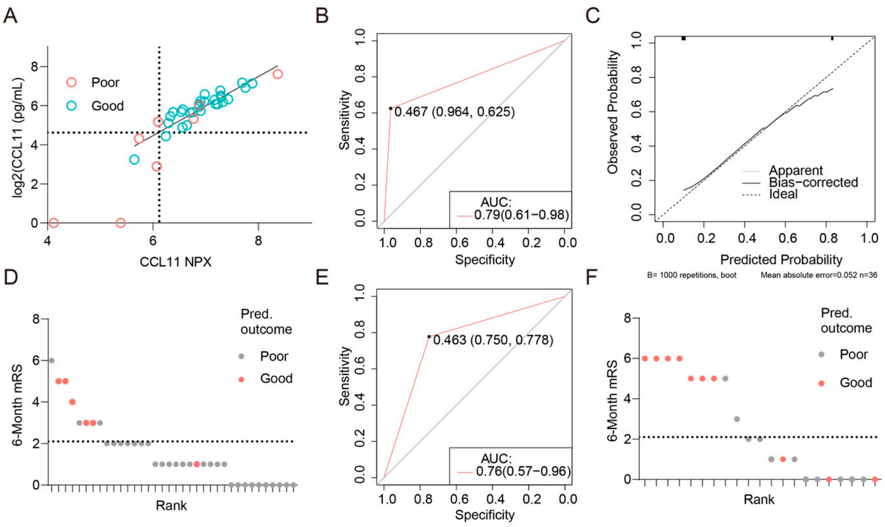

Supplementary Figure S6. Selection of biomarkers for predicting clinical outcomes in anti-NMDAR encephalitis.

(A) Fitting curves shows absolute quantifications have a linear fit with NPX values for CCL11. The dashed line represents the cutoffs. Values undetectable by ELISA are set to 0 on Y-axis.

(B) ROC curve of CCL11 logistic regression model to predict 6-month outcome in the training set. The point indicates the optimal threshold.

(C) The calibrate curve showed the goodness of fit by HL method in the training set.

(D) Waterfall diagram of the prediction for poor and good outcomes in the training set. The dashed line represents the mRS score threshold of 2.

(E) ROC curve of CCL11 logistic regression model to predict 6-month outcome in the validation set. The point indicates the optimal threshold.

(F) Waterfall diagram of the prediction for poor and good outcomes in the validation set. The dashed line represents the mRS score threshold of 2.

Supplementary Table S1. Inflammation-related proteins included

Uniprot Accession	Assay	Symbol	Description	Below LOD>0.25 (Serum):16/91	Below LOD>0.25 (CSF):32/91	Excluded
P10145	IL8	CXCL8	Interleukin-8	0.00	0.00	
P15692	VEGFA	VEGFA	Vascular endothelial growth factor A	0.00	0.00	
P01732	CD8A	CD8A	T-cell surface glycoprotein CD8 alpha chain	0.00	0.00	Yes
P80098	MCP-3	CCL7	C-C motif chemokine 7	0.03	0.31	
P39905	GDNF	GDNF	Glial cell line-derived neurotrophic factor	0.00	0.56	
Q9H5V8	CDCP1	CDCP1	CUB domain-containing protein 1	0.00	0.00	
Q9BZW8	CD244	CD244	Natural killer cell receptor 2B4	0.00	0.08	Yes
P13232	IL7	IL7	Interleukin-7	0.06	0.58	
O00300	OPG	TNFRSF11B	Tumor necrosis factor receptor superfamily member 11B	0.00	0.00	Yes
P01137	LAP TGF-beta-1	TGFB1	Transforming growth factor beta-1 proprotein	0.00	0.00	
P00749	uPA	PLAU	Urokinase-type plasminogen activator	0.00	0.00	
P05231	IL6	IL6	Interleukin-6	0.00	0.00	
Q9P0M4	IL-17C	IL17C	Interleukin-17C	0.00	0.67	
P13500	MCP-1	CCL2	C-C motif chemokine 2	0.00	0.00	
Q16552	IL-17A	IL17A	Interleukin-17A	0.14	0.14	
O14625	CXCL11	CXCL11	C-X-C motif chemokine 11	0.00	0.03	
O15169	AXIN1	AXIN1	Axin-1	0.00	0.56	Yes
P50591	TRAIL	TNFSF10	Tumor necrosis factor ligand superfamily member 10	0.00	0.03	
Q9UHF4	IL-20RA	IL20RA	Interleukin-20 receptor subunit alpha	0.53	0.33	
Q07325	CXCL9	CXCL9	C-X-C motif chemokine 9	0.00	0.00	
P28325	CST5	CST5	Cystatin-D	0.00	0.00	
P14784	IL-2RB	IL2RB	Interleukin-2 receptor subunit beta	0.00	0.28	
P01583	IL-1 alpha	IL1A	Interleukin-1 alpha	0.89	0.47	
P13725	OSM	OSM	Oncostatin-M	0.00	0.00	
P60568	IL2	IL2	Interleukin-2	1.00	0.58	
P09341	CXCL1	CXCL1	Growth-regulated alpha protein	0.00	0.00	
Q969D9	TSLP	TSLP	Thymic stromal lymphopoietin	0.50	0.86	
P13236	CCL4	CCL4	C-C motif chemokine 4	0.00	0.00	
P30203	CD6	CD6	T-cell differentiation antigen CD6	0.00	0.14	Yes
P21583	SCF	KITLG	Kit ligand	0.00	0.00	
Q14116	IL18	IL18	Interleukin-18	0.00	0.00	
Q13291	SLAMF1	SLAMF1	Signaling lymphocytic activation molecule	0.00	0.72	
P01135	TGF-alpha	TGFA	Protransforming growth factor alpha	0.00	0.00	
Q99616	MCP-4	CCL13	C-C motif chemokine 13	0.00	0.00	
P51671	CCL11	CCL11	Eotaxin	0.00	0.08	
O43557	TNFSF14	TNFSF14	Tumor necrosis factor ligand superfamily member 14	0.00	0.03	
Q9GZV9	FGF-23	FGF23	Fibroblast growth factor 23	0.28	0.58	
Q13651	IL-10RA	IL10RA	Interleukin-10 receptor subunit alpha	0.00	0.64	Yes
P12034	FGF-5	FGF5	Fibroblast growth factor 5	0.06	0.03	
P03956	MMP-1	MMP1	Interstitial collagenase	0.00	0.00	
P42702	LIF-R	LIFR	Leukemia inhibitory factor receptor	0.00	0.03	Yes
Q9NSA1	FGF-21	FGF21	Fibroblast growth factor 21	0.00	0.28	
Q99731	CCL19	CCL19	C-C motif chemokine 19	0.00	0.00	
Q13261	IL-15RA	IL15RA	Interleukin-15 receptor subunit alpha	0.06	0.44	
Q08334	IL-10RB	IL10RB	Interleukin-10 receptor subunit beta	0.00	0.00	Yes
Q8N6P7	IL-22 RA1	IL22RA1	Interleukin-22 receptor subunit alpha-1	0.86	1.00	

Q13478	IL-18R1	IL18R1	Interleukin-18 receptor 1	0.00	0.00	Yes
Q9NZQ7	PD-L1	CD274	Programmed cell death 1 ligand 1	0.00	0.00	
P01138	Beta-NGF	NGF	Beta-nerve growth factor	0.97	1.00	
P42830	CXCL5	CXCL5	C-X-C motif chemokine 5	0.00	0.03	
O14788	TRANCE	TNFSF11	Tumor necrosis factor ligand superfamily member 11	0.00	0.94	
P14210	HGF	HGF	Hepatocyte growth factor	0.00	0.00	
P29460	IL-12B	IL12B	Interleukin-12 subunit beta	0.00	0.00	
Q13007	IL-24	IL24	Interleukin-24	0.58	1.00	
P35225	IL13	IL13	Interleukin-13	0.92	1.00	
Q5T4W7	ARTN	ARTN	Artemin	0.50	0.47	
P09238	MMP-10	MMP10	Stromelysin-2	0.00	0.00	
P22301	IL10	IL10	Interleukin-10	0.00	0.50	
P01375	TNF	TNF	Tumor necrosis factor	0.00	0.28	
P55773	CCL23	CCL23	C-C motif chemokine 23	0.00	0.00	
P06127	CD5	CD5	T-cell surface glycoprotein CD5	0.00	0.00	Yes
P10147	CCL3	CCL3	C-C motif chemokine 3	0.00	0.00	
P49771	Flt3L	FLT3LG	Fms-related tyrosine kinase 3 ligand	0.00	0.00	
P80162	CXCL6	CXCL6	C-X-C motif chemokine 6	0.00	0.00	
P02778	CXCL10	CXCL10	C-X-C motif chemokine 10	0.00	0.00	
Q13541	4E-BP1	EIF4EBP1	Eukaryotic translation initiation factor 4E-binding protein 1	0.00	0.22	Yes
Q9NYY1	IL-20	IL20	Interleukin-20	0.81	0.89	
Q8IXJ6	SIRT2	SIRT2	NAD-dependent protein deacetylase sirtuin-2	0.00	0.08	
Q9NRJ3	CCL28	CCL28	C-C motif chemokine 28	0.00	0.14	
Q8NFT8	DNER	DNER	Delta and Notch-like epidermal growth factor-related receptor	0.00	0.00	Yes
P80511	EN-RAGE	S100A12	Protein S100-A12	0.00	0.28	Yes
P25942	CD40	CD40	Tumor necrosis factor receptor superfamily member 5	0.00	0.00	Yes
O95760	IL33	IL33	Interleukin-33	1.00	0.64	
P01579	IFN-gamma	IFNG	Interferon gamma	0.00	0.19	
O95750	FGF-19	FGF19	Fibroblast growth factor 19	0.00	0.03	
P05112	IL4	IL4	Interleukin-4	0.75	0.92	
P15018	LIF	LIF	Leukemia inhibitory factor	0.72	0.11	
Q99748	NRTN	NRTN	Neurturin	0.61	0.89	
P80075	MCP-2	CCL8	C-C motif chemokine 8	0.00	0.00	
Q14790	CASP-8	CASP8	Caspase-8	0.00	0.14	
O15444	CCL25	CCL25	C-C motif chemokine 25	0.00	0.17	
P78423	CX3CL1	CX3CL1	Fractalkine	0.00	0.06	
Q07011	TNFRSF9	TNFRSF9	Tumor necrosis factor receptor superfamily member 9	0.00	0.00	Yes
P20783	NT-3	NTF3	Neurotrophin-3	0.17	0.69	
O43508	TWEAK	TNFSF12	Tumor necrosis factor ligand superfamily member 12	0.00	0.00	
P78556	CCL20	CCL20	C-C motif chemokine 20	0.00	0.50	
P50225	ST1A1	SULT1A1	Sulfotransferase 1A1	0.11	0.58	Yes
O95630	STAMBP	STAMBP	STAM-binding protein	0.00	0.17	Yes
P05113	IL5	IL5	Interleukin-5	0.58	0.58	
P00813	ADA	ADA	Adenosine deaminase	0.00	0.00	
P01374	TNFB	LTA	Lymphotoxin-alpha	0.00	0.00	

Abbreviations: CSF = cerebrospinal fluid; LOD = limit of detection.

Supplementary Table S2. Association of clinical characteristics with 6-month outcome of anti-NMDAR encephalitis patients

		Good(N=28)	Poor(N=8)	p			Good (N=28)	Poor (N=8)	p
CASE score		4.00 [1.75, 12.00]	11.50 [10.00, 14.75]	0.023	CSF protein, mg/dl		29.50 [20.00, 46.50]	27.00 [20.25, 33.75]	0.493
6-month mRS		1.00 [0.00, 1.25]	3.50 [3.00, 5.00]	<0.001	CSF anti-NMDAR titer (%)	1:10	7 (25.0)	2 (25.0)	0.502
Gender (%)	F	12 (42.9)	4 (50.0)	1		1:32	16 (57.1)	3 (37.5)	
	M	16 (57.1)	4 (50.0)			1:100	5 (17.9)	3 (37.5)	
Age, years		34.00 [29.50, 40.75]	23.50 [16.50, 43.75]	0.183	Serum anti-NMDAR titer (%)	0	11 (39.3)	2 (25.0)	0.768
Inpatient, days		12.50 [10.00, 24.50]	35.00 [18.75, 70.25]	0.054		1:10	12 (42.9)	4 (50.0)	
NCU (%)	Yes	10 (35.7)	7 (87.5)	0.016		1:32	5 (17.9)	2 (25.0)	
	No	18 (64.3)	1 (12.5)			1:100	0 (0.0)	0 (0.0)	
NCU, days		0.00 [0.00, 17.00]	35.00 [16.50, 70.25]	0.004	MRI abnormal (%)	Yes	16 (57.1)	3 (37.5)	0.434
Movement disorder (%)	Yes	5 (17.9)	4 (50.0)	0.086		No	12 (42.9)	5 (62.5)	
	No	23 (82.1)	4 (50.0)		Combined autoantibody (%)	GABAR	1 (3.6)	0 (0.0)	0.652
Consciousness decline (%)	Yes	10 (35.7)	7 (87.5)	0.016		GFAP	1 (3.6)	0 (0.0)	
	No	18 (64.3)	1 (12.5)			MOG	1 (3.6)	1 (12.5)	
Speech impairment (%)	Yes	6 (21.4)	5 (62.5)	0.04		No	25 (89.3)	7 (87.5)	
	No	22 (78.6)	3 (37.5)		Combined tumor (%)	No	28 (100.0)	7 (87.5)	0.222
Seizure (%)	Yes	14 (50.0)	5 (62.5)	0.695		Teratoma	0 (0.0)	1 (12.5)	
	No	14 (50.0)	3 (37.5)		Corticosteroids pulse treatment (%)	Yes	24 (85.7)	7 (87.5)	1
Psychiatric symptom (%)	Yes	18 (64.3)	8 (100.0)	0.076		No	4 (14.3)	1 (12.5)	
	No	10 (35.7)	0 (0.0)		IVIG treatment (%)	Yes	13 (46.4)	7 (87.5)	0.053
Movement disorder (%)	Yes	9 (32.1)	4 (50.0)	0.422		No	15 (53.6)	1 (12.5)	
	No	19 (67.9)	4 (50.0)		PE treatment (%)	Yes	4 (14.3)	5 (62.5)	0.013
Autonomic nervous involved (%)	Yes	6 (21.4)	4 (50.0)	0.179		No	24 (85.7)	3 (37.5)	
	No	22 (78.6)	4 (50.0)		MMF (%)	Yes	9 (32.1)	5 (62.5)	0.217
Cognition decline (%)	Yes	15 (53.6)	4 (50.0)	1		No	19 (67.9)	3 (37.5)	
	No	13 (46.4)	4 (50.0)		Rituximab (%)	Yes	9 (32.1)	3 (37.5)	1
CSF preesure, mmH_2O		172.50 [130.00, 210.00]	175.00 [147.50, 237.50]	0.675		No	19 (67.9)	5 (62.5)	
CSF cells, 10^6 cells/l		8.00 [3.00, 28.00]	9.00 [5.00, 19.75]	0.985	Relapse during followup (%)	Yes	0 (0.0)	1 (12.5)	0.222
						No	28 (100.0)	7 (87.5)	

Consecutive or ordinal variables are shown in median [IQR]

Abbreviations: IQR = interquartile range; NCU = neurological intensive care unit; CSF = cerebrospinal fluid; IVIG = intravenous immunoglobulin; PE = plasma exchange; IVMP = intravenous methylprednisolone; NMDAR = N-methyl-D-aspartate receptor; GABAR = gamma aminobutyric acid receptor; GFAP = glial fibrillary acidic protein; MOG = myelin oligodendrocyte glycoprotein; MMF = Mycophenolate Mofetil.

Supplementary Table S3. Protein biomarkers with continuous values selected in univariate logistic regression

Severity						
Protein	Cutoff	AUC	MAX	MIN	SEN	SPE
VEGFA	11.85	0.716	13.493	8.755	0.722	0.667
IL7	2.085	0.799	3.325	-1.096	0.833	0.611
TGFB1	6.681	0.889	8.32	3.403	0.778	0.889
IL6	3.693	0.722	7.451	0.717	0.611	0.833
TNFSF10	7.651	0.812	8.853	6.413	0.611	1
CST5	6.305	0.827	7.805	4.54	0.833	0.778
IL2RB	-0.811	0.762	0.279	-1.427	0.889	0.611
CXCL1	8.756	0.673	10.477	5.207	0.611	0.778
CCL4	6.787	0.67	9.72	4.581	0.556	0.833
KITLG	8.656	0.889	9.159	5.34	1	0.611
CCL13	12.418	0.83	14.53	10.389	0.556	1
CCL11	6.892	0.873	8.367	4.118	0.889	0.778
FGF5	0.355	0.781	1.401	-0.521	0.722	0.833
MMP1	13.181	0.642	15.007	8.63	0.556	0.889
CXCL5	10.94	0.827	12.568	3.31	0.667	1
TNFSF11	2.821	0.843	5.265	0.341	0.833	0.778
HGF	8.668	0.636	11.103	6.349	0.556	0.889
IL12B	4.403	0.79	6.766	0.485	0.667	0.833
IL10	1.202	0.775	5.5	0.324	0.833	0.611
CXCL6	8.741	0.735	9.76	7.193	0.722	0.722
SIRT2	3.288	0.722	4.952	1.177	0.722	0.833
CCL8	8.985	0.91	10.458	5.666	0.889	0.833
CCL25	5.891	0.809	6.954	2.628	1	0.556
TNFSF12	6.869	0.725	8.334	5.738	0.5	1
LTA	2.663	0.904	3.966	0.922	0.889	0.833
CXCL11_CSF	-4.256	0.685	0.06	-5.71	0.889	0.444
MMP10_CSF	-1.053	0.688	1.556	-3.894	0.5	0.944
FLT3LG_CSF	1.691	0.707	3.229	0.46	0.833	0.5
CXCL10_CSF	0.765	0.701	4.648	-5.62	0.722	0.667
6-month outcome						
Protein	Cutoff	AUC	MAX	MIN	SEN	SPE
IL6	3.057	0.71	7.451	0.717	0.875	0.536
IL17C	1.179	0.732	4.104	-0.634	0.875	0.679
IL17A	-0.573	0.71	3.494	-1.775	0.75	0.786
TNFSF10	7.889	0.728	8.853	6.413	0.75	0.643
CCL11	6.173	0.754	8.367	4.118	0.625	0.964
FGF5	0.228	0.737	1.401	-0.521	0.75	0.786
MMP1	12.504	0.656	15.007	8.63	0.5	0.929
IL10	2.381	0.723	5.5	0.324	0.5	0.929
SIRT2	3.675	0.786	4.952	1.177	0.75	0.821
CCL25	4.818	0.719	6.954	2.628	0.75	0.786
CXCL11_CSF	-2.935	0.692	0.06	-5.71	0.625	0.821

Abbreviations: AUC = area under curve; AIC = Akaike information criterion; SEN = sensitivity; SPE = specificity.

Supplementary Table S4. Protein biomarkers selected in multivariate logistic regression

Severity<br>Biomarker	AUC	AIC	SEN	SPE	ACC
CST5,IL10,LTA	0.963	23.705	0.944	0.889	0.917
IL2RB,FGF5,CCL8	0.963	23.942	0.944	0.889	0.917
IL2RB,CCL11,CXCL6	0.961	26.002	0.944	0.833	0.889
IL2RB,CCL11,SIRT2	0.961	25.866	0.944	0.833	0.889
FGF5,SIRT2,CCL8	0.961	23.948	0.889	0.944	0.917
FGF5,IL10,CCL8	0.96	24.327	0.944	0.889	0.917
FGF5,IL10,LTA	0.96	24.327	0.944	0.889	0.917
CST5,IL2RB,CCL11	0.958	25.461	1	0.889	0.945
CST5,CCL8,MMP10_CSF	0.958	25.571	0.944	0.944	0.945
IL6,CST5,IL12B	0.955	25.151	0.889	0.944	0.917
CST5,IL12B,SIRT2	0.955	24.832	0.889	0.944	0.917
IL2RB,CCL11,IL12B	0.955	26.173	0.889	0.944	0.917
CCL11,IL12B,SIRT2	0.955	24.635	0.889	0.944	0.917
IL7,CST5,MMP10_CSF	0.954	25.54	0.889	0.944	0.917
TGFB1,IL6,IL2RB	0.954	26.87	0.833	0.944	0.889
CST5,IL2RB,SIRT2	0.954	26.912	0.944	0.833	0.889
CST5,IL2RB,FGF5	0.952	26.891	0.944	0.833	0.889
CST5,HGF,IL12B	0.952	27.128	0.833	0.944	0.889
CCL11,HGF,IL12B	0.952	27.027	0.833	0.944	0.889
FGF5,TNFSF11,SIRT2	0.952	26.992	0.889	1	0.945
TGFB1,IL6,CST5	0.951	27.693	0.889	0.889	0.889
TGFB1,IL6,TNFSF11	0.951	26.697	0.889	0.889	0.889
TGFB1,CST5,MMP10_CSF	0.951	27.344	0.889	0.833	0.861
IL6,CST5,IL2RB	0.951	28.289	0.944	0.944	0.945
CST5,IL12B,IL10	0.951	25.483	0.944	0.889	0.917
CCL11,CXCL6,SIRT2	0.951	27.385	1	0.778	0.889
CCL11,CXCL6,MMP10_CSF*	0.951	27.193	0.833	0.944	0.889
HGF,IL12B,CXCL6	0.951	28.783	0.833	1	0.917
IL7,CCL11,MMP10_CSF	0.949	27.194	0.889	0.889	0.889
CST5,IL2RB,IL12B	0.949	27.23	0.944	0.833	0.889
CCL11,IL12B,IL10	0.949	27.828	0.944	0.833	0.889
FGF5,IL12B,SIRT2	0.949	28.484	0.833	1	0.917
CST5,CCL11,MMP10_CSF	0.948	27.63	0.889	0.889	0.889
TGFB1,CST5,IL2RB	0.946	27.39	0.889	0.889	0.889
TGFB1,CST5,IL10	0.946	27.194	0.944	0.833	0.889
CST5,FGF5,SIRT2	0.946	27.723	0.889	0.889	0.889
CST5,FGF5,MMP10_CSF	0.946	28.035	0.833	0.889	0.861
TGFB1,TNFSF11,MMP10_CSF	0.944	28.842	0.889	0.833	0.861
CST5,CCL11,IL12B	0.944	27.605	0.889	0.889	0.889
CST5,FGF5,IL10	0.944	27.733	0.944	0.833	0.889
IL2RB,FGF5,TNFSF11	0.944	27.438	0.944	0.833	0.889
TGFB1,IL12B,MMP10_CSF	0.943	29.067	1	0.722	0.861
FGF5,HGF,IL12B	0.943	29.835	0.889	0.889	0.889
CCL11,LTA	0.941	26.343	0.833	1	0.917
CST5,CCL11,IL10	0.941	28.348	1	0.833	0.917

IL2RB,FGF5,SIRT2	0.941	28.411	0.889	0.889	0.889
TGFB1,CCL8	0.938	26.89	0.889	0.833	0.861
TGFB1,LTA	0.938	26.89	0.889	0.833	0.861
CCL11,CCL8	0.938	26.343	0.889	0.833	0.861
6-month outcome					
Biomarker	AUC	AIC	SEN	SPE	ACC
IL17C,FGF5	0.893	27.057	0.625	0.964	0.889
IL17C,CCL25	0.893	27.057	0.625	0.964	0.889
IL17C,CXCL11_CSF	0.853	31.546	0.625	0.964	0.889
IL17A,MMP1	0.902	30.04	1	0.75	0.806
IL17A,SIRT2	0.857	30.297	0.625	0.964	0.889
IL17A,CXCL11_CSF	0.848	30.282	0.75	0.786	0.778
CCL11,SIRT2	0.891	25.941	0.875	0.786	0.806
FGF5,IL10	0.848	30.04	0.875	0.714	0.750
FGF5,SIRT2	0.857	30.297	0.625	0.964	0.889
MMP1,SIRT2	0.857	29.153	0.875	0.75	0.778
MMP1,CXCL11_CSF	0.853	30.529	0.875	0.75	0.778
IL10,CCL25	0.848	30.04	0.875	0.714	0.750
IL10,CXCL11_CSF	0.799	33.119	0.75	0.75	0.750
IL17A,MMP1,CXCL11_CSF	0.935	24.684	0.875	0.929	0.917

*Represents selected biomarkers

Abbreviations: AUC = area under curve; AIC = Akaike information criterion; SEN = sensitivity; SPE = specificity; ACC = accuracy.

Supplementary Table S5. Demographic and clinical characteristics of the validation cohort

	Groups	N=21		Groups	N=21
CASE (mean (SD))		8 [3,11.5]	CSF_Cell (median [IQR])		9.00 [4.00, 24.00]
CASE severity (%)	Sereve	11 (52.4)	protein_CSF (median [IQR])		29.70 [26.00, 41.00]
	Mild	10 (47.6)	CSF_aNMDAR_titre (%)	0	2 (9.5)
6-monthmRS (median [IQR])		2.00 [0.00, 5.00]		1:10	7 (33.3)
6-month6 outcome (%)	Good	12 (57.1)		1:32	6 (28.6)
	Poor	9 (42.9)		1:100	6 (28.6)
Gender (%)	F	12 (57.1)	Serum_aNMDAR_titre (%)	0	9 (42.9)
	M	9 (42.9)		1:10	5 (23.8)
Age (median [IQR])		33.00 [24.00, 61.00]		1:32	6 (28.6)
Inpatient (median [IQR])		18.00 [13.00, 44.00]		1:100	1 (4.8)
ICU (%)	Yes	12 (57.1)	Combined_autoantibody (%)	AQP4	1 (4.8)
	No	9 (42.9)		MOG	4 (19.0)
ICU_days (median [IQR])		14.00 [0.00, 42.00]		No	16 (76.2)
Consciousness (%)	Yes	12 (57.1)	Combined_tumor (%)	0	19 (90.5)
	No	9 (42.9)		Teratoma	2 (9.5)
Respiratory_support (%)	Yes	6 (28.6)	Corticoid (%)	Yes	19 (90.5)
	No	15 (71.4)		No	2 (9.5)
Speech (%)	Yes	10 (47.6)	IVIG (%)	Yes	12 (57.1)
	No	11 (52.4)		No	9 (42.9)
Seizure (%)	Yes	13 (61.9)	PE (%)	Yes	10 (47.6)
	No	8 (38.1)		No	11 (52.4)
Psychosis (%)	Yes	16 (76.2)	MMF (%)	Yes	17 (81.0)
	No	5 (23.8)		No	4 (19.0)
Movement (%)	Yes	2 (9.5)	MRI_abnormal (%)	Yes	11 (52.4)
	No	19 (90.5)		No	10 (47.6)
Autonomic_nervous (%)	Yes	7 (33.3)	Relapse_during_followup (%)	Yes	1 (4.8)
	No	14 (66.7)		No	20 (95.2)
Cognition (%)	Yes	13 (61.9)	Rituximab (%)	Yes	15 (71.4)
	No	8 (38.1)		No	6 (28.6)
CSF_preesure (median [IQR])		180.00 [150.00, 230.00]			

Consecutive or ordinal variables are shown in median [IQR]

Abbreviations: IQR = interquartile range; NCU = neurological intensive care unit; CSF = cerebrospinal fluid; IVIG = intravenous immunoglobulin; PE = plasma exchange; NMDAR = N-methyl-D-aspartate receptor; GABAR = gamma aminobutyric acid receptor; GFAP = glial fibrillary acidic protein; MOG = myelin oligodendrocyte glycoprotein. MMF = Mycophenolate Mofetil.
